# Effect of Vitamin D in HN9.10e Embryonic Hippocampal Cells and in Hippocampus from MPTP-Induced Parkinson’s Disease Mouse Model

**DOI:** 10.3389/fncel.2018.00031

**Published:** 2018-02-07

**Authors:** Samuela Cataldi, Cataldo Arcuri, Stéphane Hunot, Carmen Mecca, Michela Codini, Maria E. Laurenti, Ivana Ferri, Elisabetta Loreti, Mercedes Garcia-Gil, Giovanna Traina, Carmela Conte, Francesco S. Ambesi-Impiombato, Tommaso Beccari, Francesco Curcio, Elisabetta Albi

**Affiliations:** ^1^Department of Pharmaceutical Sciences, University of Perugia, Perugia, Italy; ^2^Department of Experimental Medicine, University of Perugia, Perugia, Italy; ^3^Institut du Cerveau et de la Moelleépinière, Inserm U 1127, CNRS UMR 7225, UPMC Univ Paris 06, UMR S 1127, Sorbonne Universités, Paris, France; ^4^Division of Anatomic Pathology and Histology, Department of Experimental Medicine, School of Medicine and Surgery, University of Perugia, Perugia, Italy; ^5^Department of Biology, University of Pisa, Pisa, Italy; ^6^Interdepartmental Research Center Nutrafood, Nutraceuticals and Food for Health, University of Pisa, Pisa, Italy; ^7^Dipartimento di Area Medica, University of Udine, Udine, Italy

**Keywords:** Parkinson disease, N-cadherin, embryonic hippocampal cells, neurofilaments, vitamin D

## Abstract

It has long been proven that neurogenesis continues in the adult brains of mammals in the dentatus gyrus of the hippocampus due to the presence of neural stem cells. Although a large number of studies have been carried out to highlight the localization of vitamin D receptor in hippocampus, the expression of vitamin D receptor in neurogenic dentatus gyrus of hippocampus in Parkinson’s disease (PD) and the molecular mechanisms triggered by vitamin D underlying the production of differentiated neurons from embryonic cells remain unknown. Thus, we performed a preclinical *in vivo* study by inducing PD in mice with MPTP and showed a reduction of glial fibrillary acidic protein (*GFAP) and vitamin D receptor* in the dentatus gyrus of hippocampus. Then, we performed an *in vitro* study by inducing embryonic hippocampal cell differentiation with vitamin D. Interestingly, vitamin D stimulates the expression of its receptor. Vitamin D receptor is a transcription factor that probably is responsible for the upregulation of microtubule associated protein 2 and neurofilament heavy polypeptide genes. The latter increases heavy neurofilament protein expression, essential for neurofilament growth. Notably N-cadherin, implicated in activity for dendritic outgrowth, is upregulated by vitamin D.

## Introduction

During the last 25 years epidemiological and basic research studies have demonstrated the involvement of Vitamin D3 (VD3) in the brain physiopathology (Millet et al., 2014). VD3 has neurotrophic and neuroprotective actions and influences neurotransmission and synaptic plasticity (Groves et al., 2014), playing a role in various neurological diseases, i.e., multiple sclerosis (Sundström and Salzer, 2015). Recently, accumulating evidence showed that VD3 plays a protective role in neurocognitive disorders (Schlögl and Holick, 2014; Wood and Gupta, 2015), by acting in areas where memory resides, as hippocampus of animals affected with Alzheimer’s disease (AD) (Anastasiou et al., 2014; Durk et al., 2014). Many observations showing that cognitive impairment is present not only in AD but also in later stages of Parkinson’s disease (PD) were reported (Riedel et al., 2008; Samat et al., 2017). Hippocampus includes Cornu Ammonis (CA) differentiated in CA1, CA2, CA3, and CA4 areas filled with densely packed pyramidal cells. CA4 is often called the hilus or hilar region and it is considered as a part of the dentate gyrus (DG) (Amaral, 1978). Unlike the CA1 and CA3, the neurons of C4 receive inputs from the granule cells in the DG that therefore contains hilus and the fascia dentata (Amaral, 1978). The DG of hippocampus is a region of the adult brain where neurogenesis takes place and plays a role in the formation of new memories (Nakashiba et al., 2012). It has long been proven that neurogenesis continues in the adult brains of mammals in the subgranular zone (SGZ) of the DG (Cameron et al., 1993; Eriksson et al., 1998) due to the presence of stem cell-like granule progenitor cells with radial morphology (Seki et al., 2007; Kriegstein and Alvarez-Buylla, 2009; Liu et al., 2010). These granule progenitor cells express the glial fibrillary acidic protein (GFAP) astrocyte marker (Fukuda et al., 2003; Garcia et al., 2004; Seki et al., 2007; Kriegstein and Alvarez-Buylla, 2009; Liu et al., 2010; Morrens et al., 2012), as well as general neural stem cell markers, such as brain lipid-binding protein (BLBP), Nestin, Sox1, and Sox2 (Suh et al., 2007; Hodge and Hevner, 2011; Hsieh, 2012; Venere et al., 2012). Experimental ablation of GFAP^+^cells prevents the production of new neurons, indicating that the removal of GFAP^+^ cells reduces the ability of the germinal niche to regenerate (Garcia et al., 2004). These studies suggest that hippocampal granule cells are generated from GFAP-expressing progenitor cells. NSCs in the SGZ give rise to mature neurons of the granular layer involved in learning and memory (Tashiro et al., 2006).

An increasing number of studies demonstrated that VD3 receptor (VDR) and enzymes involved in VD3 metabolism are expressed in the central nervous system, particularly in the areas of hippocampus (Langub et al., 2001; Gezen-Ak et al., 2013). VDR expression is altered in some neurodegenerative disorders. In fact, reduction of VDR mRNA levels in AD as compared to Huntington hippocampus has been reported (Sutherland et al., 1992). Stress (Ji et al., 2014) and AD (Sutherland et al., 1992) change hippocampal VDR expression. In addition, hypovitaminosis D results in neurological dysfunction that might explain part of the cognitive disorders both in the general population and in AD patients (Annweiler, 2014) by acting not only in the progression of neurodegenerative diseases, but also as an aggravating co-factor (Millet et al., 2014). Regarding the role of VD3 in PD, most researchers have focused their attention on the effect of VD3 in calcium metabolism and consequently on bone disorders and fractures (Sato et al., 2001, 2005, 2011; Invernizzi et al., 2009; Jones et al., 2010; Iwamoto et al., 2012; van den Bos et al., 2013; Wang et al., 2016; Tassorelli et al., 2017). An association between low blood level of VD3 and PD (reviewed by Lv et al., 2014; Rimmelzwaan et al., 2016) as well as VDR polymorphism and PD (Niu et al., 2015) have been described. The role of VD3 has been studied in cellular and animal models of PD, specifically in the substantia nigra and striatum. VD3 inhibits microglial activation, protecting dopaminergic neurons (Kim et al., 2006) and increases the expression of glial cell line-derived neurotrophic factor (Sanchez et al., 2009), thus facilitating neuroprotection (Orme et al., 2016). *In vitro* studies, performed in embryonic hippocampal cells (HN9.10e cell line) have shown that VD3 supplementation up to 100 nM stimulates VDR expression, reduces mitosis and cell division with accelerated neurite outgrowth and increased NGF production in the hippocampus (Brown et al., 2003; Marini et al., 2010). To do it VD3 translocates from the cytoplasm to the nucleus where it localizes in nuclear lipid microdomains that act as platform for transcription process (Bartoccini et al., 2011). After serum withdrawal, the dose of VD3 had to be increased to obtain embryonic hippocampal cell differentiation (Bartoccini et al., 2011).

Whether there VDR expression is altered in the hippocampus and in particular in the neurogenic zones of hippocampus of PD patients with or without cognitive impairment is presently unknown. The aim of the study was to investigate the perturbation of VDR in neurogenic DG of hippocampus. In the present work we performed a preclinical study by inducing PD in mice with 1-methyl-4-phenyl-1,2,3,6-tetrahydropyridine (MPTP) (Cataldi et al., 2016, 2017). To highlight the molecular events occurring during VD3-induced differentiation, we draw on immunohistochemical, immunofluorescent, and molecular biology data to show how VD3 induces the formation of neurites in HN9.10e cells.

## Materials and Methods

### Reagents

Dulbecco’s modified Eagle’s medium (DMEM), bovine serum albumin (BSA), dithiothreitol, phenylmethylsulfonylfluoride (PMSF) were obtained from Sigma Chemical, Co. (St. Louis, MO, United States); VD3 was obtained from DBA Italia (Segrate, Milan, Italy); anti-GFAP antibody was obtained from Dako, Agilent (Santa Clara, CA, United States), anti-N-cadherin, anti- peroxisome proliferator-activated receptor gamma (PPARγ), anti-VDR from Elabscience (Houston, TX, United States) and anti-beta actin antibodies were obtained from Santa Cruz Biotechnology, Inc. (Santa Cruz, CA, United States); anti-neuron specific enolase (NSE) and anti-NF200 antibodies were from NOVOCASTRA Laboratories, Ltd. (Newcastle, United Kingdom). For research involving biohazards, biological select agents and reagents, standard biosecurity safety procedures were carried out.

### Animals and Treatments

Ten- to twelve-week-old male C57BL/6 J mice, weighing 25–30 g (CERJ, France), were used. Mice were kept in a temperature-controlled room (23 ± 1°C) under a 12-h light/dark cycle and had *ad libitum* access to food and water, as previously reported (Cataldi et al., 2016). Animals were maintained and treated according to ethical regulations and guidelines (Guide for the Care and Use of Laboratory Animals; NIH publication number 85-23; revised 1985) and the European Communities Council Directive 86/609/EEC. Experimental protocols were performed according to the French national chart for ethics of animal experiments (articles R 214-87 to 126 of the “Code rural”) and received approval from the ethical committee number 5 “Charles Darwin” and from the ICM animal care and use committee. Groups of mice (*n* = 5) received MPTP under an acute protocol and control mice (*n* = 5) received an equivalent volume of 0.9% NaCl solution, as previously reported (Cataldi et al., 2016). After removal brains were post-fixed overnight in fresh 4% paraformaldehyde (PFA)/phosphate-buffered saline (PBS) solution, cryoprotected with 30% sucrose in 0.1 M PB, and frozen in isopentane (-30°C). Free-floating brain sections (20 μm thick) encompassing the hippocampus were prepared using a freezing microtome (Microm, Germany). Samples of three different sections (8 μm thick) were collected and used for immunofluorescence analysis.

### Cell Culture and Treatments

Immortalized hippocampal neurons HN9.10e (kind gift of Dr. Kieran Breen, Ninewells Hospital, Dundee, United Kingdom) were cultured as previously reported (Marini et al., 2010). VD3, dissolved in absolute ethanol as vehicle at the 100 nM physiological concentration (Vieth, 2006), was added to the cultures for 48 h; in control samples only absolute ethanol was added (Marini et al., 2010). The cells were used in part for immunofluorescence and immunocytochemical analysis, and in part for RT-PCR, and immunoblotting analysis.

### Immunofluorescence

The cryostat sections were incubated over night with 3% (w/v) BSA, 1% (w/v) glycine in PBS to block non-specific sites, as previously reported (Arcuri et al., 2002). The morphometric analysis of hippocampus was performed by using Image Focus software. Then sections were incubated with anti-GFAP or anti-VDR primary antibodies diluted 1:100 in 3% (w/v) BSA in PBS for 1 h, washed three times in 0.1% (v/v) Tween-20 in PBS and twice in PBS, incubated with tetramethylrhodamineisothiocyanate (TRITC)-conjugated anti-rabbit IgG for 1 h, diluted 1:50 in 3% (w/v) BSA in PBS and washed as above. The diamidino-2-phenylindole (DAPI) nuclear counterstain was used. The samples were mounted in 80% (w/v) glycerol, containing 0.02% (w/v) NaN_3_ and *p*-phenylenediamine (1 mg/ml) in PBS to prevent fluorescence fading. The antibody incubations were done in a humid chamber at room temperature. HN9.10e were cultured for 48 h to evaluate N-cadherin and PPARγ and for 4 days to evaluate GFAP, VDR, with specific antibodies. After treatment with primary antibodies performed as reported above, TRITC-conjugated anti-rabbit IgG was used for GFAP and VDR and fluorescein isothiocyanate (FITC)-conjugated anti-rabbit IgG was used for N-cadherin and PPARγ were used as above reported. Nuclei were counterstained with DAPI. Fluorescent analysis was performed on a DMRB Leika epi-fluorescent microscope equipped with a digital camera. In the tissue, the positive granules of VRD were counted. The intensity of immunofluorescence of VDR, N-cadherin and PPARγ in HN9.10e cells was evaluated with Scion Image.

### Reverse Transcription Quantitative PCR (RTqPCR)

HN9.10e were cultured for 48 h for RTqPCR analysis. Total RNA was extracted from control and VD3-treated cells by using RNAqueous^®^-4PCR kit (Ambion, Inc., Austin, TX, United States). Samples were treated and RTqPCR was performed according to Codini et al. (2015) to study the gene expression of microtubule-associated protein 2 (MAP2, Hs1103243_g1) and neurofilament heavy polypeptide (NEFH, Hs00912472), genes, insulin gene enhancer protein (ISL1, Hs01099686_m1). Glyceraldehyde-3-phosphate dehydrogenase (GAPDH, Hs99999905-m1) was used as housekeeping gene.

### Immunocytochemistry

HN9.10e were cultured for 48 h for immunocytochemical analysis. Bond Dewax solution was used toremove paraffin from sections before rehydration and immunostaining on the Bond automated system (Leica Biosystems Newcastle, Ltd., United Kingdom) as previously reported (Albi et al., 2012). Immunostaining for NSE and neurofilament heavy protein (NF200) detection was performed by using specific antibodies and Bond Polymer Refine Detection – Leica Biosystems (Newcastle, Ltd., United Kingdom). The observations were performed by using inverted microscopy EUROMEX FE 2935 (ED Amhem, Netherlands) equipped with a CMEX 5000 camera system (100x magnification). The intensity of immunostaining was evaluated. The findings were classified as no reactive cells, low positive cells, medium positive cells, and strong positive cells. Only the strong positive cells were considered for quantification, as previously reported (Marini et al., 2010).

### Electrophoresis and Western Immunoblotting

HN9.10e were cultured in the presence of VD3 for 48 h for immunoblotting analysis. Total protein concentration was determined spectrophotometrically at 750 nm and about 40 μg proteins were submitted to SDS-PAGE electrophoresis in 10% polyacrylamide slab gel. Immunoblotting was performed as previously reported (Cataldi et al., 2014) by using anti-N-cadherin, anti-PPARG, andanti-beta-actin primary specific antibodies. The apparent molecular weight of the proteins was calculated according to the migration of molecular size standards. The area density of the bands was evaluated by densitometry scanning and analyzed with Scion Image (Cataldi et al., 2014).

### Statistical Analysis

Three experiments were performed in duplicate for each analysis. Data were expressed as mean ± SD and *t*-test was used for statistical analysis.

## Results

### *In Vivo* Study of Vitamin D3 Receptor in Hippocampus in Parkinson’s Disease

**FIGURE 1:**
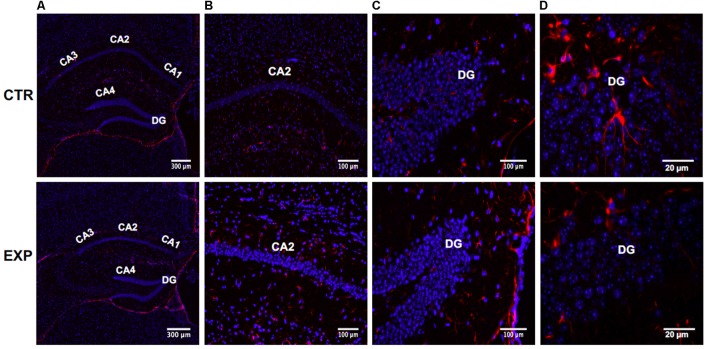
Hippocampus in normal (CTR) and MPTP-induced Parkinson’s disease (PD) mice (Exp). CA, Cornu Ammonis; differentiated into CA1, CA2, CA3, and CA4 areas filled. DG, dentatus gyrus. The images represent the merged signals with DAPI signals (blue) in the nuclei and immunolabeling with anti-GFAP (red). **(A)** 10x magnification; **(B,C)** 20x magnification; **(D)** 100x magnification oil immersion.

We used the MPTP-induced PD mouse model. VDR was localized in neurogenic zones of DG in hippocampus of adult mice (Amaral, 1978). Downregulation of hippocampus VDR was reported in AD (Sutherland et al., 1992; Gezen-Ak et al., 2013). Instead, VDR has never been analyzed in PD although some patients have memory deficiency as AD patients (Foster et al., 2017). Therefore we sought to study the specific cell type expressing VDR in DG of hippocampus in MPTP-induced PD mouse model. To assess the expression and distribution of VDR, we first stained sections of brain containing hippocampus with marker for radial astrocytes (GFAP) counterstained with DAPI. As shown in **Figures 1A,B**, hippocampal total volume is decreased in PD mice, reflecting a loss of volume in all hippocampal subfields. The morphometric analysis shows that the length of hippocampus is 7.2 ± 1.0 μm in control and 5.3 ± 1.2 in PD mice and its thickness is 2.05 ± 0.04 in control and 0.52 ± 0.01 in PD mice. CA1, CA2, and CA3 appeared clearly thinner. The DG has a different form. The upper and lower parts of DG, as well as the corner delimited by them appear thinner, with the upper terminal part truncated. The distance between upper and lower parts of DG is increased, making the CA4 area larger. These morphological differences were not accompanied by an increase in the number of cells, indicating an absent or very moderate inflammatory response in PD mice.

GFAP^+^-astrocytes show a different distribution and appear less stellate in both CA2 (**Figure 1B**) and DG (**Figure 1C**). GFAP appears less expressed in hippocampus DG of MPTP-treated mice compared to the healthy control, probably underlining a reduced presence of stem cells. In fact, GFAP^+^ cells in the healthy hippocampus DG have been evidenced and represent quiescent or activated stem cells. However, these preliminary results need further studies.

**FIGURE 2:**
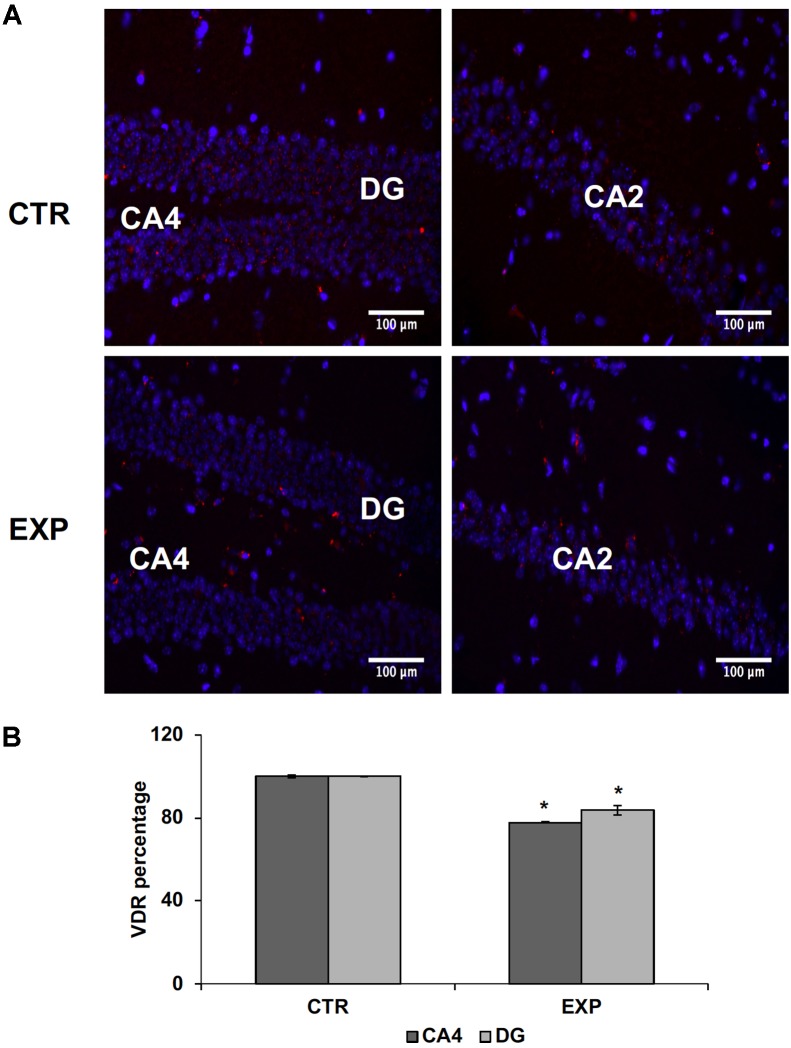
Hippocampus in normal (CTR) and MPTP-induced PD mice (Exp). CA2, and CA4, Cornu Ammonis areas; DG, dentatus gyrus. **(A)** The images represent the merged signals with DAPI signals (blue) in the nuclei and immunolabeling with anti-VDR (red), 20x magnification. **(B)** The positive granules of VDR were counted in CA4 and DG. Data represent the percentage of positive granules in experimental samples respect to control samples and they are expressed as mean ± SD of three independent experiments. ^∗^*P* < 0.05.

In addition, externally to the DG, GFAP expression in PD hippocampus is increased, compared to the control, reflecting the presence of moderate astrogliosis without cell proliferation (**Figures 1B–D**). These changes were correlated with reduced levels of VDR previously associated to cognitive disorders (Gezen-Ak et al., 2013). In hippocampal tissue of PD mice, VDR protein (red staining) appears strongly decreased in both CA2 and DG; it is particularly evident in the corner of DG (**Figures 2A,B**).

### *In Vitro* Study of the Vitamin D3 Effect on Embryonic Hippocampal Cells: Neurite Elongation

**FIGURE 3:**
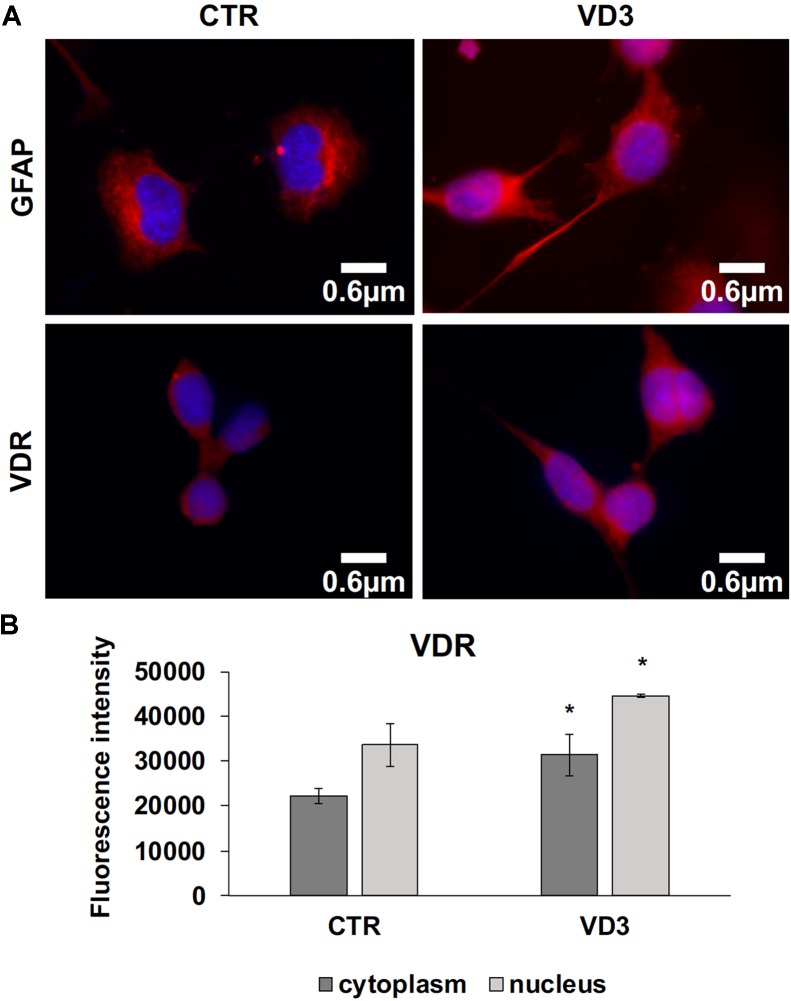
Embryonic hippocampal HN910.e cells (CTR, control cells) and cultured with VD3. **(A)** The images represent the merged signals with DAPI signals (blue) in the nuclei and immunolabeling with anti-GFAP (red) or VDR (red), 100x magnification oil immersion. **(B)** VDR immunofluorescence signal intensity in the cytoplasm and nuclei was analyzed before merging. Data are expressed as mean ± SD of three independent experiments. ^∗^*P* < 0.05.

**FIGURE 4:**
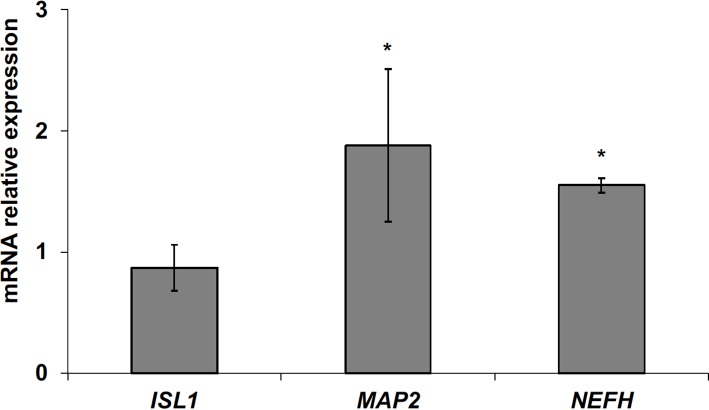
Effect of vitamin D3 on insulin gene enhancer protein (*ISL1*), microtubule-associated protein 2 (*MAP2*) and neurofilament heavy polypeptide (*NEFH*) expression. RTqPCR analysis was performed in control and VD3-treated HN9.10e cells, by using GAPDH as housekeeping gene. In ordinate, mRNA relative expression = mRNA of VD3-treated cells/mRNA of control cells. Data are expressed as the mean ± SD of three independent experiments performed in three PCR replicates. ^∗^*P* < 0.05.

**FIGURE 5:**
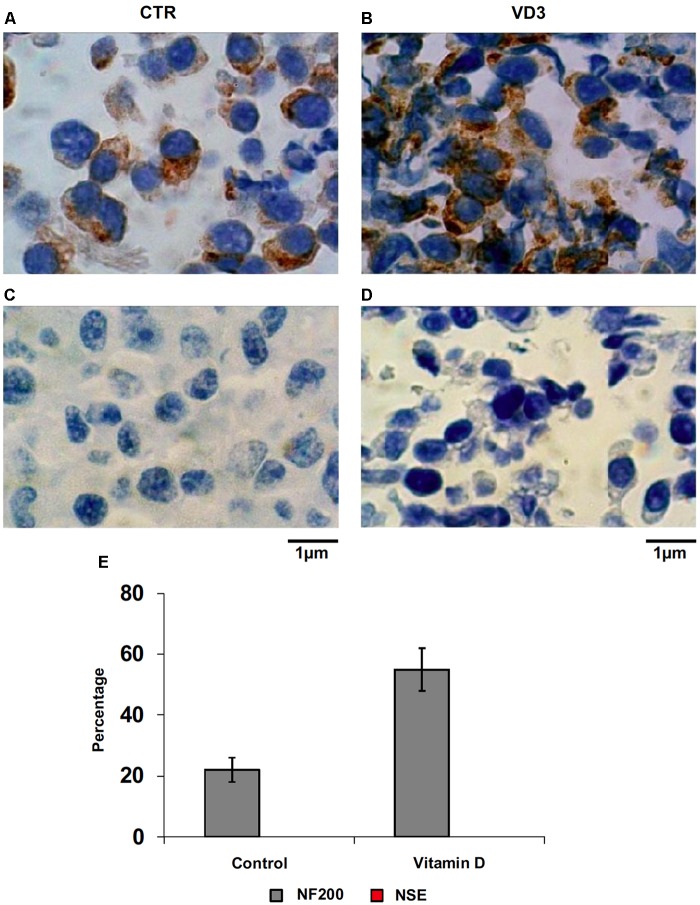
Immunocytochemical analysis of Neurofilament 200 kDa (NF200) and neuron specific enolase (NSE). **(A)** NF200 in control HN9.10e cells; **(B)** NF200 in VD3-treated HN9.10e cells; **(C)** NSE in control HN9.10e cells; **(D)** NSE in VD3-treated HN9.10e cells; **(A–D)** 40x magnification. **(E)** Data are expressed as percentage of total cells that resulted highly stained (positive cells) and represent the mean ± SD of three independent experiments performed in duplicate. ^∗^*P* < 0.05.

**FIGURE 6:**
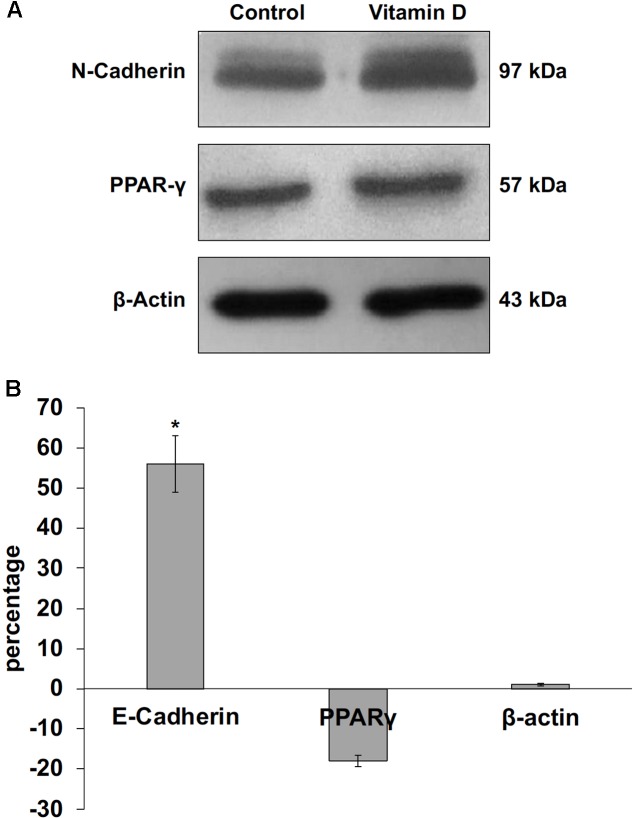
Immunoblotting analysis of N-cadherin, peroxisome proliferator-activated receptor gamma (PPARγ) and beta-actin in control and VD3-treated HN9.10e cells. **(A)** The position of the 97 kDa for N-cadherin, 57 kDa for PPARG and 43 kDa for beta-actin was evaluated in relation to the position of molecular size standards. **(B)** The area density was quantified by densitometry scanning and analysis with Scion Image. Data are expressed as percentage variation of VD3-treated HN9.10e cells compared with control HN9.10e cells and represent the mean ± SD of three independent experiments. ^∗^*P* < 0.05.

**FIGURE 7:**
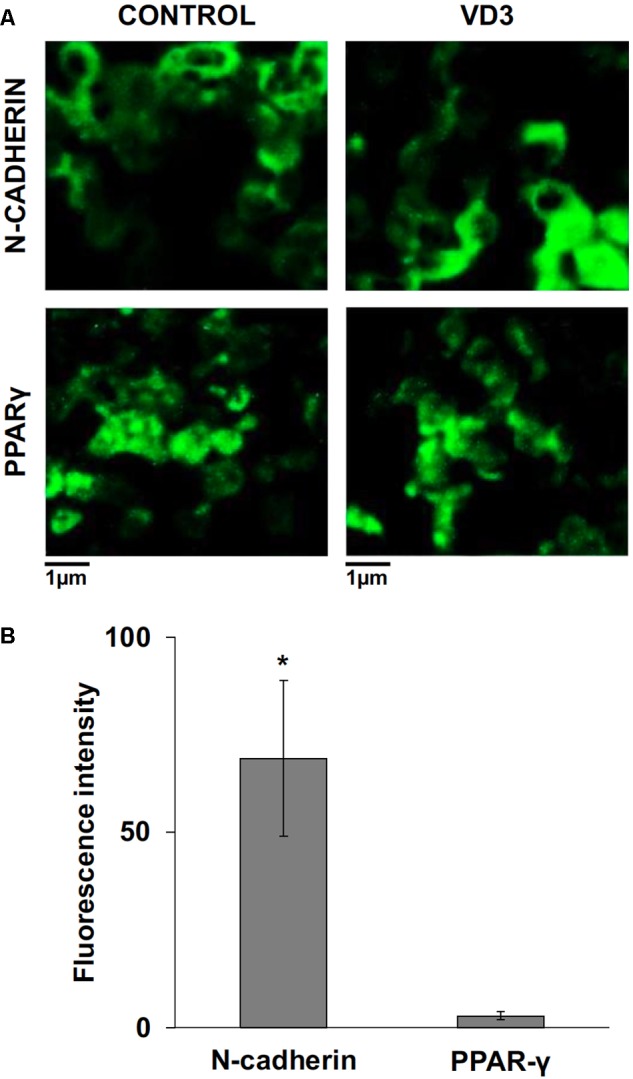
Immunofluorescence analysis of N-cadherin and peroxisome proliferator-activated receptor gamma (PPARγ) in control and vitamin D3-treated HN9.10e cells after 48 h of culture; **(A)** N-cadherin and PPARγ immunofluorescence, CTR and VD3 treated cells, 40x magnification. **(B)** N-cadherin and PPARγ immunofluorescence signal was analyzed as reported in materials and methods. Data are expressed as percentage increase of signal in VD3-treated HN9.10e cells compared with control HN9.10e cells and represent mean ± SD of three independent experiments. ^∗^*P* < 0.05.

The data obtained in *in vivo* experiments, indicated a simultaneous reduction in expression and location changes of VDR^+^ and GFAP^+^ hippocampal neurogenic cells. Our previous results had demonstrated, by immunoblotting analysis, that the treatment with VD3 of HN9.10e cells was able to upregulate nuclear VDR (Marini et al., 2010). Thus, we chose to highlight the effect of VD3 on VDR by immunofluorescence counterstained with DAPI because this result might be very important for the correlation with *in vivo* study. **Figures 3A,B** shows that VDR is overexpressed by VD3 in both cytoplasm and nuclei Then, since adult hippocampal neural stem/progenitor cells express GFAP both when they originate neurons and astrocytes (Venere et al., 2012), we wondered whether HN9.10e embryonic hippocampal cells in culture might express GFAP and in case of positive response whether the expression of the protein could change after VD3 treatment. To this end we cultured the cells with VD3 and we performed the immunostaining with GFAP counterstained with DAPI. In embryonal HN9.10e control cells, the GFAP positivity was detected in cytoplasm, whereas HN9.10e VD3 treated cells also showed GFAP positivity in neurites (**Figure 3**). In this case, VD3 could induce differentiation. Moreover, the formation of neurites during VD3-induced HN9.10e cell differentiation was previously demonstrated (Marini et al., 2010) but molecules involved in this process remained unknown. Thus, to determine the contribution of VD3 to this process, we treated HN9.10e cells for 48 h to study the gene expression of MAP2 and NEFH, genes for putative neurite elongation proteins. ISL1, a protein involved in neuronal development in the retina and in the striatonigral via (Lu et al., 2014), was used for comparison. We found an upregulation of MAP2 and NEFH genes without change of ISL1 (**Figure 4**), suggesting a correlation between VD3 treatment and MAP2-NEFH expression. Since NEFH encodes 200–220 kDa neurofilament heavy proteins, the result prompted us to carry out an immunocytochemical study of NF200 protein. Anti-NSE antibody, a generic marker of mature neurons (Marangos et al., 1979) was used for comparison. The results showed that VD3 increases 2.5-fold the NF200 specific staining (**Figures 5B,E**) in comparison with control sample (**Figure 5A**). No NSE staining is present in control orVD3-treated cells (**Figures 5C,D**). Given the central role of N-cadherin in mediating interaction between neuron precursor cells and in regulating further differentiation (Yagita et al., 2009), it became important to define its content and localization in HN9.10e cells without or with VD3 treatment. Moreover PPARγ as neuroprotective molecule (Thouennon et al., 2015) was considered. For these studies, immunoblotting and immunofluorescence techniques were utilized. We found that control and VD3-treated cells present immunoreactivity in correspondence to the bands with apparent molecular weight of 97 kDa for N-cadherin, and 57 kDa for PPARγ (**Figure 6A**). VD3 treatment increases 57% N-cadherin band intensity without important change of PPARγ (**Figure 6B**). Accordingly, the immunofluorescence signal of N-cadherin increases after VD3 treatment, in particular among nearby cells; no difference of signal is evident for PPARγ (**Figures 7A,B**).

## Discussion

Glial cells undergo a rapid and transient expansion from progenitor cells within the first several weeks of postnatal life, which coincides with increasing neuronal activity in the hippocampus (Catalani et al., 2002; Traina, 2017). In the present work, an *in vitro* and *in vivo* preclinical study was designed to highlight: (1) the behavior of VDR in the DG of hippocampus in MPTP-treated mice and (2) the molecular mechanisms involved in the neurite development during VD3-induced embryonic hippocampal cell differentiation. To our knowledge, this is the first study to describe reduction of VDR in the DG in a mice model of PD. In DG, GFAP^+^ cells represent NSCs, i.e., radial glial or progenitors cells that continuously gives rise to mature neurons and astrocytes (Venere et al., 2012; Gebara et al., 2016). These cells have been involved in memory formation and in other functional roles (Tashiro et al., 2006; Hsu, 2007; Traina, 2017). Our preliminary results show in DG of mice with MPTP-induced PD a lower number of GFAP^+^ cells in comparison with control mice. We could speculate a minor neurogenesis in PD-induced mice. Although we used only GFAP staining as marker to detect NSCs, the result is interesting and should be confirmed by using other specific markers. This result correlates with a simultaneous reduction of VDR^+^ cells in DG. Outside of the DG, the GFAP expression in both control and PD hippocampus is quite evident with differentiated positive astrocytes that appear slightly more numerous in PD mice probably reflecting a moderate astrogliosis, without cell proliferation. In this context, a previous study has evidenced increased GFAP levels, detected by ELISA, in frontal cortex and striatum but not in hippocampus of mice with MPTP-induced PD compared to control (Domenger et al., 2012). A recent work has shown that in MPTP mice, hippocampal astrogliosis detected by GFAP immune-labeling is evident (Manocha et al., 2017). This study does not investigate the GFAP localization in the various regions of the hippocampus. Our study is in agreement with this work, since a GFAP increase has been highlighted. We have also tried to study this increase in GFAP immunoreactivity in the hippocampal regions. We have evidenced a greater number of GFAP^+^ cells externally to the DG. This datum has been interpreted as modest astrogliosis. While the decreased number of GFAP^+^ cells in the DG of MPTP mice compared to control has been hypothesized to be due to a lower presence of stem cells *tout court*. We have used GFAP labeling for its double capacity to detect reactive astrocytes (astrogliosis) and stem/progenitor cells. However, this is only an interesting preliminary study that will need to be supported by quantitative data and the use of other markers.

More importantly, this is the first report describing molecules involved in VD3-induced HN9.10e cell differentiation. In fact we show that VD3 upregulates MAP2 that encodes for the homonym protein localized along microtubules (Takemura et al., 1992) and NEFH that encodes for NF200 protein, both proteins being associated to the axon and dendritic shape maintenance (Giust et al., 2014). Furthermore, VD3 increases NF200 protein as a consequence of *NEFH* up-regulation. It is known that microtubules, microfilaments and neurofilaments play a crucial role in maintaining structure and function of neurites in mature neurons (Gentil et al., 2015; Traina, 2016). On the other hand we show that VDR is upregulated by VD3, confirming previous data (Marini et al., 2010). VDR is a ligand-activated transcription factor (Zenata and Vrzal, 2017) and VD3 links VDR in nuclear lipid microdomains that act as platform for RNA transcription (Albi and Villani, 2009). Another new finding of our study is that HN9.10e cells express GFAP and it is evident along processes during VD3-induced differentiation in agreement with the notion that in the adult hippocampus, GFAP-expressing neural progenitors give rise to immature neurons via early intermediate progenitors expressing both GFAP and neuronal markers (Liu et al., 2010). Moreover, in control cells cytoplasmic GFAP staining demonstrates that they represent stem or progenitor cells (Zhang and Jiao, 2015) since GFAP expression in embryonic hippocampal granule progenitor cells has been detected, similarly to adult hippocampal neural progenitor cells (Seki et al., 2014). It is in agreement with previous studies demonstrating that during the embryonic stages, both hippocampal and neocortical progenitor cells arise from a similar neurogenic zone of the cortex, the ventricular zone (Tabata et al., 2012; Taverna et al., 2014; Ostrem et al., 2017). Previous studies have demonstrated that spontaneous differentiation of human and mouse embryonic stem cells in culture may occur (Heo et al., 2005; Sathananthan and Trounson, 2005). Likewise, GFAP^+^ HN9.10e cells may undertake spontaneous differentiation after 7–10 days of culture (data not shown), reflecting their nature of stem cells and the ability to give rise to a more differentiated progeny. However, in HN9.10e cells this phenomenon is uncommon. Finally, GFAP expression in HN9.10e cells is predictive of their neural stem cell characteristics. It is worth to note that in adult hippocampal neurogenesis, GFAP is not a stationary marker of neural stem cells, but it detects a range of immature cells, from active radial glia to type-2a, but not 2ab, intermediate progenitor cells (Zhang and Jiao, 2015).

Adult neurogenesis has many common characteristics with embryonal neurogenesis and parallelisms are possible (Kriegstein and Alvarez-Buylla, 2009; Fuentealba et al., 2015). In agreement with the developmental neurogenesis, the primary progenitors for the continual generation of neurons in postnatal animals correspond to cells with glial characteristics: radial glial cells (RGCs) or astrocytes.

The structure of the adult DG is characterized by a typical lamination composed by a variety of neuronal cells. The DG formation is a complex process that requires RGCs for normal development (Barry et al., 2008). In the mouse hippocampus GFAP+ radial processes are first detected on E16 (Woodhams et al., 1981).

These cells subsequently undergo embryonic and postnatal cytoarchitectonic reorganization and give rise to adult hippocampus. In the adult hippocampus, RG-like cells are preserved, i.e., stem cells, and are responsible to produce both new neurons and astrocytes during life (Kempermann et al., 2004). GFAP^+^ HN9.10.e cells thus represent RGCs or intermediate progenitors cells with stemness property. During VD3-induced differentiation these cells undergo morphological and biochemical changes, evidenced by GFAP re-organization, as *in vivo* DG maturation. These results suggest that VD3-VDR may be involved not only in regulating adult hippocampal neurogenesis, but also in embryonic and postnatal maturation of DG. In addition, our data show that VD3 increases N-cadherin that belongs to the type I subfamily of cadherins and plays a role in maintaining synaptic contact (Seong et al., 2015). Given the findings of this study and published work on relationship VD3-neurological disorders, the results suggest the involvement of VD3-VDR in PD. In summary, this study demonstrates reduction in VDR and loss of differentiation ability of DG stem cells in PD and at the same time the capability of VD3 to stimulate VDR and consequently allow growth of neurites by inducing hippocampus embryonic cells to a mature state. This implies that the VD3-VDR is an important regulator of cell differentiation.

## Author Contributions

In this study, EA designed the work and prepared the manuscript. SC and CA performed the principle experiments and prepared the images. CM, SH, IF, EL, MC, and TB performed the experiments. ML, MG-G, CC, and GT contributed to interpretation of data. FA-I and FC contributed to analyzing the data and gave financial support. All authors read and approved the final manuscript.

## Conflict of Interest Statement

The authors declare that the research was conducted in the absence of any commercial or financial relationships that could be construed as a potential conflict of interest.
